# Photodetector based on Vernier-Enhanced Fabry-Perot Interferometers with a Photo-Thermal Coating

**DOI:** 10.1038/srep41895

**Published:** 2017-01-31

**Authors:** George Y. Chen, Xuan Wu, Xiaokong Liu, David G. Lancaster, Tanya M. Monro, Haolan Xu

**Affiliations:** 1 Laser Physics and Photonic Devices Laboratories, School of Engineering, University of South Australia, Mawson Lakes, South Australia 5095, Australia; 2 Future Industries Institute, University of South Australia, Mawson Lakes, South Australia 5095, Australia

## Abstract

We present a new type of fiber-coupled photodetector with a thermal-based optical sensor head, which enables it to operate even in the presence of strong electro-magnetic interference and in electrically sensitive environments. The optical sensor head consists of three cascaded Fabry-Perot interferometers. The end-face surface is coated with copper-oxide micro-particles embedded in hydrogel, which is a new photo-thermal coating that can be readily coated on many different surfaces. Under irradiation, photons are absorbed by the photo-thermal coating, and are converted into heat, changing the optical path length of the probing light and induces a resonant wavelength shift. For white-light irradiation, the photodetector exhibits a power sensitivity of 760 pm/mW, a power detection limit of 16.4 μW (i.e. specific detectivity of 2.2 × 10^5^ cm.√Hz/W), and an optical damage threshold of ~100 mW or ~800 mW/cm^2^. The response and recovery times are 3.0 s (~90% of change within 100 ms) and 16.0 s respectively.

Photodetectors are amongst the most widely used photonic technologies, which can be found in light meters, cameras, medical scanners and analyzers, smoke detectors, security systems, high-end vehicles, optical communication systems, remote controls, printer scanners, surveying instruments, and so forth. Photodetectors can operate using a wide range of different sensing mechanisms^1^,^2^,^3^,^4^,^5^,^6^,^7^,^8^,^9^,^10^, including photo-emission, photo-electric, photo-voltaic, photo-thermal and photo-chemical. Most photodetectors reported in the literature feature an electrical sensor head with an output in the form of an electrical signal. This gives rise to a serious problem in environments that are subject to electro-magnetic interference (EMI), which can distort the electrical signal as it propagates through electrical components and wires. One solution is to use an optical sensor head by extending a standard photodetector with an optical fiber connected to a multimode ball-lens fiber that collects light. The optical sensor head with an output in the form of an optical signal, and with its waveguide being dielectric makes it immune to electro-magnetic interference (EMI) and suitable for electrically sensitive environments^11^,^12^. To avoid signal distortion, the probe light source and the photo-electric conversion module can be located remotely from the optical sensor head in the measurement environment. However, strong irradiation can damage the photo-electric conversion module if it is any type other than photo-thermal. Employing a neutral-density filter is not ideal, because it not only introduces interference, but also increases the power detection limit. Photo-thermal based technologies tend to exhibit higher optical-damage thresholds (e.g. 30 kW commercial thermopiles) but also higher detection limits than those of the other sensing mechanisms^13^,^14^. However, if the photo-thermal type is chosen for the photo-electric conversion module rather than the optical sensor head, the guided light is likely to be too weak for detection.

To address the sequence of problems, we present for the first time a photodetector with an optical sensor head based on the photo-thermal effect, and a photo-electric conversion module based on the photo-electric effect to process the optical signal into an electrical signal outside the measurement environment. Potential applications include high-power medical and industrial lasers, power meters and spacecraft^15^. With an interferometric design^16^, the optical sensor head is more sensitive than absorption-based designs^17^. A fiber-bulk architecture rather than an all-fiber design allows the optical sensor head to: (a) capture all the light from a typical collimated laser beam (e.g. <4 mm spot diameter), and thus simplifies power measurements because focusing is not needed; and (b) enable the bonding of photo-thermal coatings with large particles beyond tens of microns in diameter. A comparison between our photodetector and the state-of-the-art thermal-based photodetectors is presented in Table 1. Note that the units of power sensitivity differs from device to device depending on the sensing mechanism. The specific detectivity (D*) gives the figure of merit regardless of the technology. Although this photodetector does not surpass the others with some of the parameters, its immunity against EMI is unique and its detection area is sufficiently large.

## Results and Discussion

### Sensor-head fabrication

**Figure 1:**
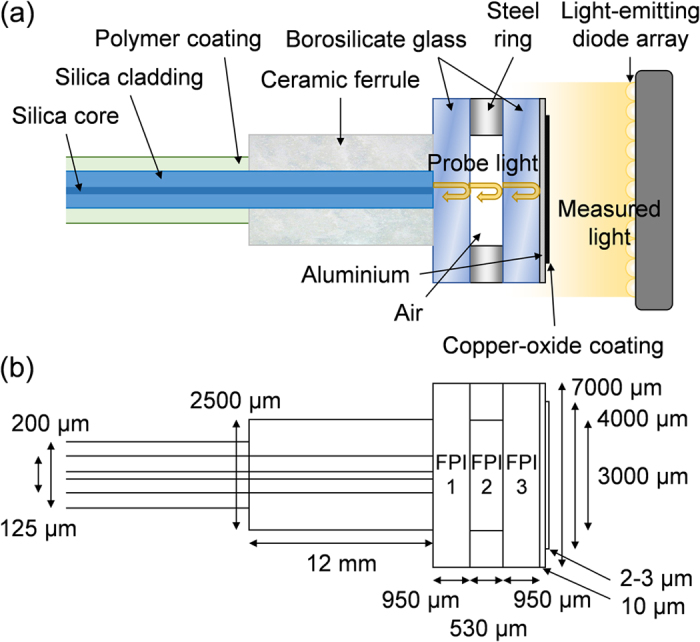
Schematic of the optical sensor head of the photodetector showing (**a**) the labeled components, and (**b**) the dimensions. FPI: Fabry-Perot interferometer.

The design of the optical sensor head is shown in Fig. 1(a), with the dimensions labeled in Fig. 1(b). The variations are ±5 μm for the layer thicknesses, except the copper-oxide coating. A single-mode optical fiber (i.e. SMF-28) was flat cleaved and inserted into a ceramic ferrule. A square slab of borosilicate glass was cut and polished before being attached to the end-face of the ferrule and fixed with ultraviolet-curable epoxy resin (i.e. NOA-61)^18^. This was followed in the same manner by a steel ring, another borosilicate glass layer and a thin layer of aluminium. The first glass layer forms the first reference Fabry-Perot interferometer (FPI)^19^ due to the refractive-index (RI) difference at the glass-glass and glass-air interfaces. The air gap created by the metal ring establishes the second reference FPI, due to the glass-air and air-glass interfaces. Photodetectors that measure power need to be able to dissipate or convert the photon-induced phonon energy as quickly as possible, in order to facilitate a fast response and accurate measurement. Therefore, a ring made of metal is not only geometrically flat, but also helps to rapidly dissipate heat through thermal conduction. On the contrary, photodetectors designed to measure energy must do the opposite, and thus retain heat for as long as possible. The second glass layer creates the sensing FPI, due to the air-glass and glass-metal interfaces. The reflectance spectrum of the probe light is used as the output signal, and thus the reflective metal layer is necessary to prevent the probe light from entering the adjacent photo-thermal coating on the metal layer surface. This coating is designed to respond only to the measured light source. Otherwise, a fourth FPI is created with random absorption caused by the thermal expansion of its micro-particles. EMI does not interact with the metal ring or layer in any way that affects the sensor head. The surface area of the sensor head should be made according to the irradiation beam diameter (e.g. 1–30 mm).

**Figure 2:**
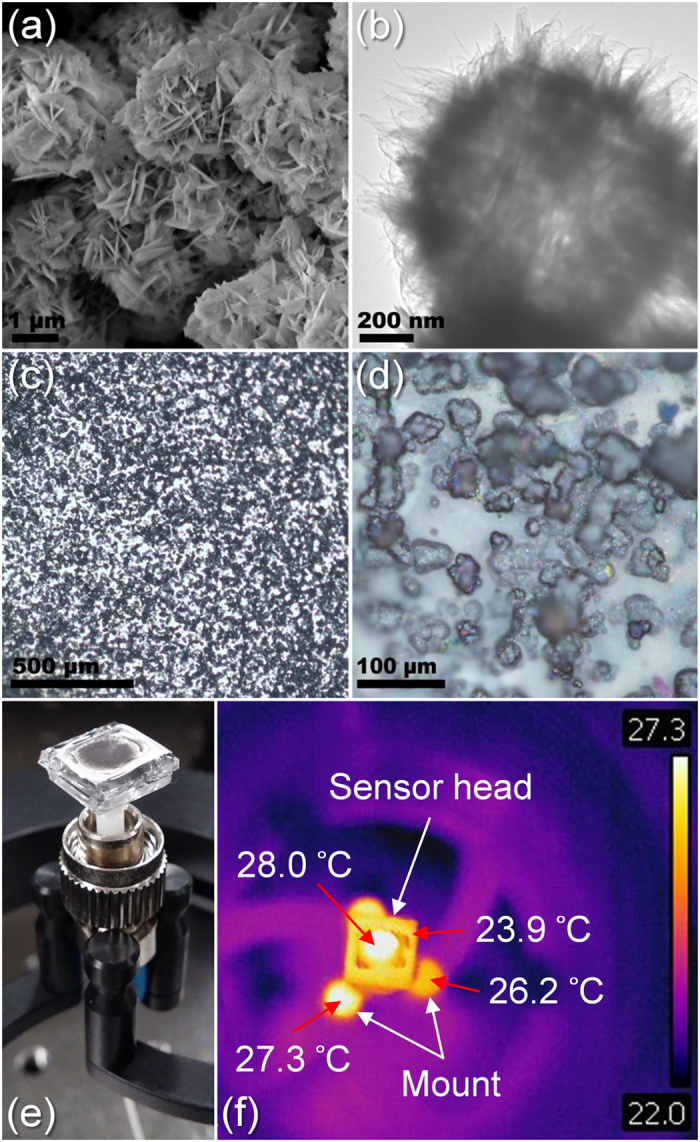
(**a**) Scanning electron microscope (SEM) image and (**b**) transmission electron microscope (TEM) image of the flower-like CuO micro-particles. Microscope images of the copper-oxide coating under (**c**) 5×, and (**d**) 20× magnification. (**e**) Photograph of the sensor head. (**f**) Infrared image of the sensor head when irradiated by 2.6 mW of white light.

The photo-thermal effect^20^ originates from the conversion of photon energy into phonon energy (i.e. heat). It induces a temperature increase on the coating surface. Currently, the most studied photo-thermal effect stems from the surface plasmon resonance of noble metals such as Pd^21^ and Au^22^. However, these noble metals are expensive, limiting their range of applications. Recently, it was found that CuO is an excellent photo-thermal material^23^,^24^. Compared with those noble metals, CuO is abundant and cheap, which is evident in Table S1. Flower-like hollow micro-particles with diameters of 1.9 ± 0.1 μm that are composed of CuO nanoleaves were synthesized shown in Fig. 2(a,b). The flower-like hollow structure of CuO can enhance light trapping and absorption via multiple reflections within the structure^25^, and thus promotes the photo-thermal conversion efficiency. As shown in Fig. S1, the CuO micro-particles appear black in colour. The ultraviolet-visible (UV-Vis) spectrum confirms high absorption (i.e. 97.5%) of light ranging between 200–700 nm wavelength. The CuO micro-particles are then embedded in agarose hydrogel, and finally bonded onto the surface of the metal layer. The result is a new type of photo-thermal coating of 4 mm diameter and 2–3 μm thickness shown in Fig. 2(c,d) and Fig. S2. Upon irradiation, the build-up of heat within the sensing FPI is partially suppressed from transferring to the two reference FPIs by the air gap. As a result, the change in the optical path length via thermal expansion and the thermo-optic effect is much more significant for the sensing FPI compared with the two reference FPIs. With this configuration, the three cascaded FPIs can utilize the Vernier effect^26^,^27^ (i.e. envelope tracking of superimposed resonances) to enhance the resonant wavelength shift (Δ*λ*_*R*_).

### Photo-thermal characterization

**Figure 3:**
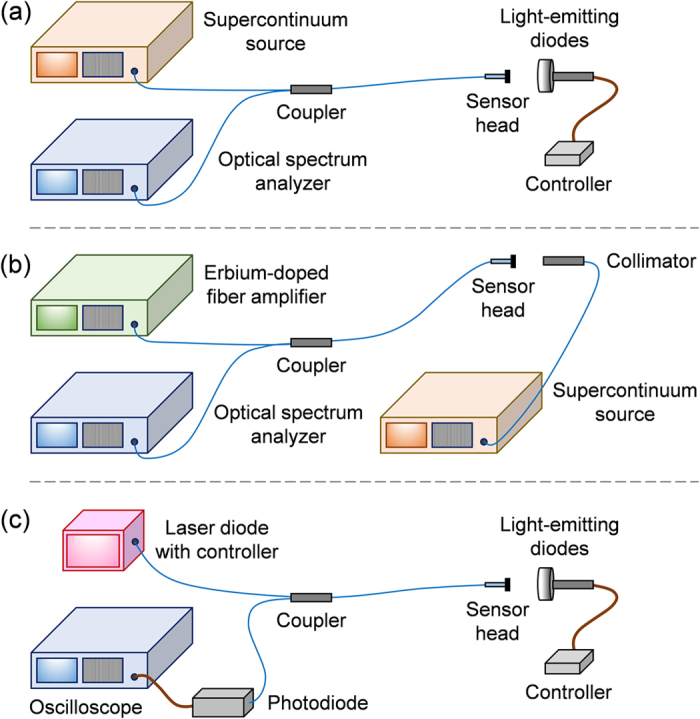
Schematic of the experimental setup of the photodetector for (**a**) white-light irradiation characterization, (**b**) selective-wavelength irradiation characterization, and (**c**) temporal characterization.

The sensor head was probed with unpolarized light from a supercontinuum source (SCS) (i.e. YSL SC-5, YSL AOTF-PRO) shown in Fig. 3(a), delivering a continuous range of wavelengths from 1305 nm to 1330 nm, which is within the uniform spectral region of the SCS. All the optical fibers used are standard telecommunications fibers (i.e. SMF-28). The reflectance spectrum from the sensor head was diverted by a fused fiber coupler, then collected and analyzed by an optical spectrum analyzer (OSA) (i.e. Ando AQ-6315E) that serves as the photo-electric conversion module. The OSA was set to HIGH2, 1001 sampling points, 31.25 Hz sampling rate and 2 running averages. A circular array of unpolarized white light-emitting diodes (LED) was used as the measured light source, which only generates visible light and emits negligible heat. Not shown in Fig. 3 is a reference thermopile detector (i.e. Newport 843-R) that was used to measure the incident optical power.

**Figure 4:**
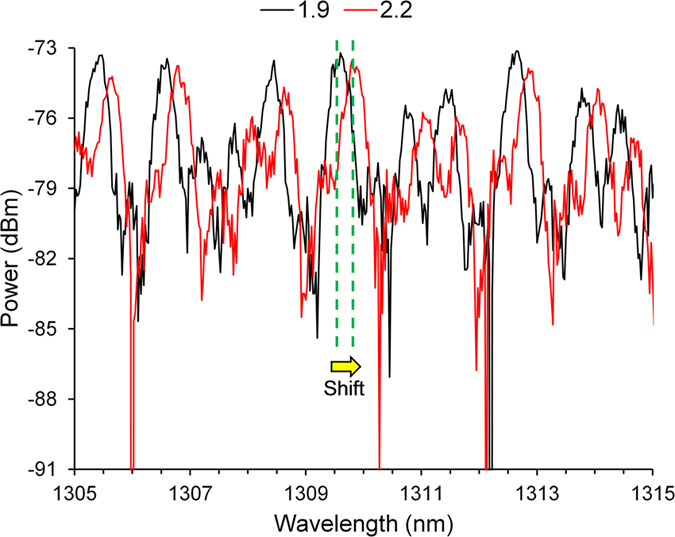
Example resonant wavelength shift when the incident optical power from the white-light LED array (i.e. probed by the SCS) was increased from 1.9 mW to 2.2 mW.

To test the performance of this photodetector, different levels of optical power were irradiated onto the sensor head by changing the drive current to the LEDs, and Δ*λ*_*R*_ exemplified in Fig. 4 was recorded by the OSA. Since the spot diameter of the incident light (i.e. ~100 mm) is larger than that of the sensor head (i.e. 4.0 mm) and reference thermopile detector (i.e. 9.5 mm), the incident optical power measured by the reference thermopile detector was scaled based on the ratio between the detection area of the sensor head and reference thermopile. The sensor head was initially annealed at the highest level of incident optical power to ensure that any water content in the epoxy resin was removed, so that the sensor head will not experience further permanent changes during the measurements. The delay between each measurement was 5 min, to ensure that the temperature of the sensor head has stabilized. The temperature increase on the surface of the sensor head can be observed by an infrared camera (i.e. FLIR TG165) to be 6.9 °C, as shown in Fig. 2(f). The reason why one contact of the mount had a temperature close to that of the sensor head is because it received extra light from reflections off the central structure. However, the mount temperature does not affect the performance of the photodetector.

In Fig. 4, there is a complex interplay of the three cascaded FPIs, because the different pairing of the four interfaces can behave as six FPIs exploiting the Vernier effect. Owing to the short coherence length (i.e. as short as 5.8 μm) of the white-light LEDs (i.e. ~200 nm bandwidth at full width at half maximum), only some of the adjacent FPIs contribute to the output signal. The coherence length (*L*_*C*_) is the distance in the direction of wave-front propagation within which the amplitude and phase of the wave is well defined and predictable (i.e. >1/e interference visibility). For a Gaussian-shaped emission spectrum, it is given by^28^:


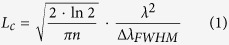


where *n* is the RI of the medium (e.g. *n* = 1.50 at 1.3 μm wavelength for borosilicate glass), and *λ* is the wavelength of light.

As a result, the resonance pattern contains both small and large features, with extinction ratio of some resonances exceeding 20 dB. The contributors are the free spectral ranges (FSR) of the first and second reference FPIs, being 0.6 nm and 1.7 nm respectively. The FSR of a single FPI can be calculated from the following expression^29^:


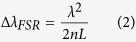


where *L* is the etalon length of the FPI.

A resonance was nominated and tracked to measure the steady-state Δ*λ*_*R*_. For a single FPI, it is governed by the expression^30^:


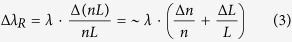


where Δ*n* is the thermo-optic coefficient (e.g. n = ~4 × 10^−6^ RIU/°C for borosilicate glass^31^), and Δ*L*/*L* is the linear coefficient of thermal expansion (e.g. 3.3 × 10^−6^/°C for borosilicate glass^32^).

**Figure 5:**
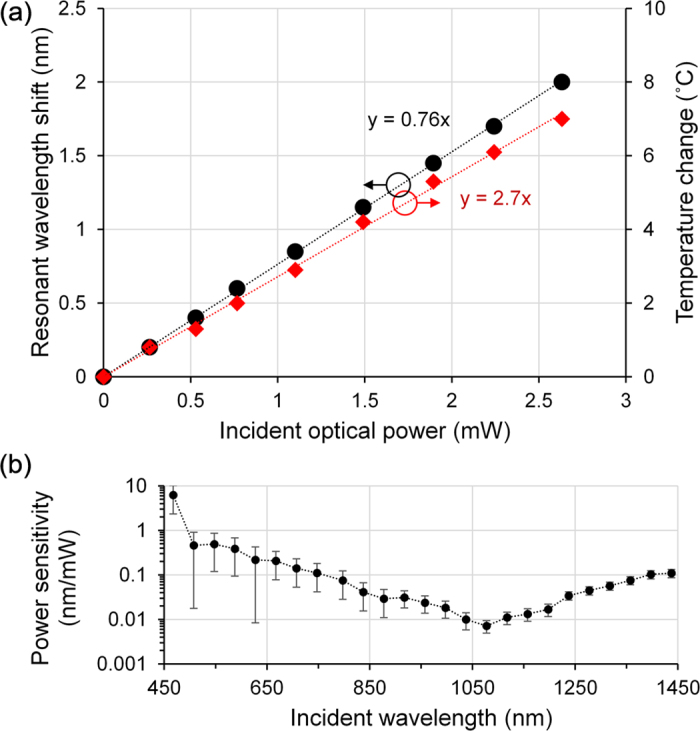
(**a**) Resonant wavelength shift and local temperature as a function of the optical power of white light from the LED array (i.e. probed by the SCS); and (**b**) wavelength-resolved power sensitivity when the measured light source is the SCS (i.e. probed by the EDFA). Line fittings are applied to determine the sensitivities.

After plotting the measured data in Fig. 5(a), the power sensitivity relating the incident optical power and the corresponding Δ*λ*_*R*_ is approximately linear at ~760 pm/mW in the measured range. The linear conversion factor can be used to provide a readout of the optical power from the wavelength data. From the infrared images, if the first-stage conversion factor is 2.7 °C/mW, the second-stage conversion factor is ~280 pm/°C. As expected from the Vernier effect, Δ*λ*_*R*_ of the three cascaded FPIs is one order of magnitude larger than that of a single FPI (e.g. 7.8 pm/°C at 1.3 μm wavelength was simulated for a single glass layer). The power sensitivity can be increased by: (a) selecting a photo-thermal coating with a higher conversion-efficiency; (b) choosing a glass with higher thermo-optic and thermal expansion coefficients; and (c) optimizing the etalon length of the FPIs.

To investigate Δ*λ*_*R*_ of different wavelengths, the experimental setup of the photodetector was modified according to Fig. 3(b), such that an unpolarized erbium-doped fiber amplifier (EDFA) (i.e. Keopsys KPS-BT2-C-33-PM-BO-FA) was used to probe the sensor head with a wavelength range of 1540–1560 nm, and the SCS irradiated the sensor head. The impact of changing both the probe light source and measured light source is a change in the extinction ratio of the observed resonances, and a scaling of the power sensitivity. This is because: (a) all the measured light sources emit unpolarized light which lead to roughly the same ratio of s- and p-polarization reflections; (b) as the probe light sources exhibit different spectral bandwidths, the different temporal coherences generate resonances with different extinction ratios; (c) although the 1305–1330 nm wavelength range of the SCS guides two modes in SMF-28 (i.e. single mode for ~1550 nm), the higher-order mode is relatively weak and can be neglected; and (d) Eq. 3 indicates that longer probe wavelengths experience linearly proportionally larger shifts (i.e. fewer wavelengths in the cavity, each one needs a larger change to compensate for the cavity change), and in this case the gain factor is 1.18. Since the spot diameter of the incident light (i.e. 1.0 mm) is smaller than that of the sensor head (i.e. 4.0 mm) and reference thermopile detector (i.e. 9.5 mm), the incident optical power measured by the reference thermopile detector was not scaled.

The wavelength-resolved power sensitivity plotted in Fig. 5(b) reveals that the sensor head is more efficient at converting light into heat at the lower end of the visible spectrum, which is also the case with standard thermopile detectors^33^. This is deduced from the larger Δ*λ*_*R*_ per mW of optical power, which implies a higher temperature rise and thus higher phonon energy was converted from the same amount of photon energy. The power sensitivity gradually decreases towards the near-infrared spectrum, until it reaches a local minimum around 1078 nm. Beyond this point, the power sensitivity slowly increases. The average (i.e. mean) power sensitivity of the visible spectrum at 1.3 nm/mW is shows a high degree of similarity to that of the white-light irradiation at 1.1 nm/mW multiplied by the gain factor of 1.18. The wavelength-resolved power sensitivity can potentially be flatter with the right choice or blend of photo-thermal micro-particles and embedding medium. With the smallest wavelength resolution of the OSA being 25.0 pm, the wavelength detection limit is 12.5 pm. The error bars are too small to be visible in Fig. 5(a). The error bars in Fig. 5(b) were obtained from dividing the wavelength detection limit by the incident optical power to give the error-equivalent power sensitivity. The longer lower error-bars at 508 nm and 628 nm are due to a combination of lower incident-power, logarithmic scale and lower power-sensitivity.

For the following calculations, the white-light data was used to represent the average performance of the photodetector in the visible spectrum. Dividing the wavelength detection limit of 12.5 pm by the power sensitivity of 760 pm/mW, the power detection limit is deduced to be 16.4 μW. This is only a few times higher than that of standard thermopile detectors^14^. D* is 2.2 × 10^5^ cm.√Hz/W, calculated from dividing the square root of the detection area-frequency bandwidth product by the power detection limit. The initial temperature sensitivity calculated from Fig. 5(a) is 2.7 °C/mW. The material with the lowest degradation-temperature of 260 °C is the ultra-violet-curable epoxy resin. Therefore, dividing the temperature limit by the temperature sensitivity yields an optical damage threshold of ~100 mW. Given that the photo-thermal coating area is 12.6 mm^2^, the intensity equivalent is ~800 mW/cm^2^. The damage threshold can be increased beyond 815 °C by using a ceramic-metal epoxy resin^34^ or glass soot^35^,^36^, enabling an optical damage threshold of ~300 mW or ~2400 mW/cm^2^. This will be closer to those of commercial products^14^. There is a trade-off between power sensitivity and damage threshold. For instance, using a photo-thermal coating with a higher conversion-efficiency (i.e. higher power-sensitivity) will result in the sensor head heating up to the damage threshold at a lower level of the incident optical power (i.e. lower optical-damage threshold).

### Temporal characterization

**Figure 6:**
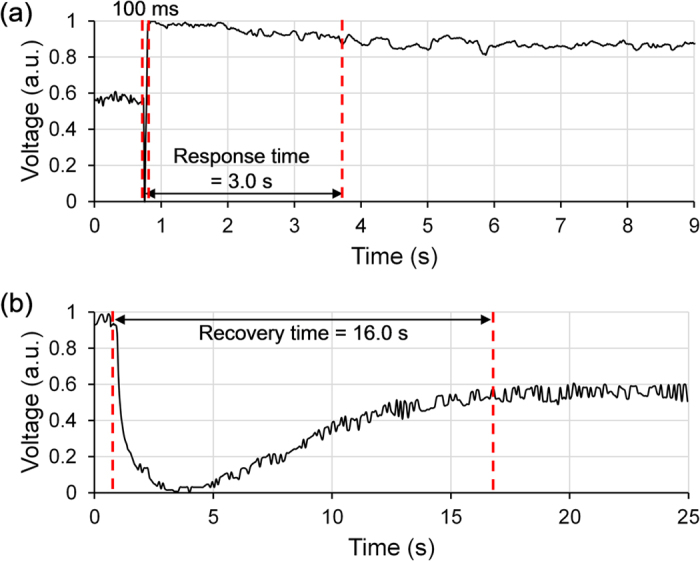
(**a**) Response time measurement from 0 mW to 1.5 mW produced by the white-light LED array (i.e. probed by the laser diode); and (**b**) recovery time measurement from 1.5 mW to 0 mW.

The temporal characteristics of the optical sensor head shown in Fig. 6 were measured by modifying the experimental setup of the photodetector shown in Fig. 3(c). A 1550 nm narrow-linewidth laser diode (i.e. Qphotonics QFBGLD-1550-10, Thorlabs CLD1015) was used as the probe light source. The white-light LED array was used as the measured light source, producing an incident optical power of 1.5 mW. A photodiode (i.e. Thorlabs PDB450C) connected to an oscilloscope (i.e. Rigol DS6104) replaced the OSA. The warm-up time of 3.0 s corresponds to the response time, though ~90% of the change occurred within the first 100 ms. This is of the same order of magnitude as that of standard thermopile detectors^37^. The cool-down time of 16.0 s corresponds to the recovery time.

Generally, the photo-thermal response starts very quickly before slowing down. Firstly, this is caused by the process of reaching thermal equilibrium where there is an increasing slowdown with time. Secondly, it is due to Newton’s law of cooling where hotter objects lose heat more rapidly than colder objects. Therefore, warming up is the quickest at the beginning due to the smallest temperature difference and thus the largest positive rate difference between heat gain and heat loss. Cooling down is also the quickest at the beginning due to the largest temperature difference and thus the highest rate of heat loss through conduction, convection and irradiation. The response time is significantly shorter than the recovery time, because heat transfer through irradiation-conduction is faster than conduction-convection. For higher incident optical powers, the change in response time will be negligible (i.e. faster heat gain offset by faster heat loss), but the recovery time will slightly increase (i.e. higher phonon energy takes longer to dissipate).

Specifically for this optical sensor head, the rapid introduction of heat within the first 100 ms of the response time is predominately caused by: (a) the thin layers of the copper-oxide/hydrogel and aluminium (i.e. ~10 μm) allows fast propagation of phonons; and (b) the irradiation/heat-induced increase of free charge-carriers increases the thermal conductivity^38^ of the copper oxide, producing a very short transient response resembling the first half of passive Q-switching^39^ (i.e. giant pulse formation from laser cavities). Essentially, the first wave of phonons will arrive at the region of light with the subsequent train of faster and faster phonons joining in at more or less the same time. However, this mechanism also works the other way by slowing down heat dissipation, which prolongs the recovery time.

There is also a trade-off between power sensitivity and response/recovery time. For example, a thicker photo-thermal coating (i.e. appears darker) is superior at trapping light and retaining heat, but the larger thermal mass prolongs the time required for the sensor head to attain thermal equilibrium. Also, using a conductive ring instead of a dielectric ring for the air gap quickens the temporal characteristics, at the cost of pulling more heat away from the sensing FPI.

Although Δ*λ*_*R*_ are more readily tracked using the conjunction of a broadband light source and an OSA, a low-cost alternative is possible. For example, a narrow-linewidth laser diode (e.g. InGaAsP) and a photodiode (e.g. InGaAs) can be paired up with a low-cost digital processor (e.g. Raspberry Pi) to track Δ*λ*_*R*_. Fringe ambiguities can be avoided by using the predetermined pattern of the operating spectral range as a reference.

This photodetector can be evolved to provide spatial information, by simply replacing the single-mode optical fiber with a multi-core single-mode optical fiber. The probing light in each core are uncoupled and can be analyzed separately to give information about the measured light at 2-dimensional positions across the detection area.

## Conclusions

We have proposed and experimentally demonstrated a new type of photodetector that employs an optical sensor head using the photo-thermal effect. The optical sensor head can even operate in environments with high levels of electro-magnetic interference. For white-light irradiation, a power sensitivity of 760 pm/mW, a power detection limit of 16.4 μW (i.e. specific detectivity of 2.2 × 10^5^ cm.√Hz/W), and an optical damage threshold of ~100 mW or ~800 mW/cm^2^ were achieved. Response and recovery times of 3.0 s (~90% of change within 100 ms) and 16.0 s respectively were measured. These promising preliminary results can be further optimized in terms of the power sensitivity, wavelength-resolved power sensitivity, temporal response, power detection limit and optical damage threshold with the aforementioned modifications in material and geometry. The immunity against electro-magnetic interference enables the photodetector to be used in applications where existing photodetector technologies are unsuitable. Furthermore, this photodetector can be modified with a multi-core optical fiber for spatially resolved measurements.

## Experimental section

### Materials

CuSO_4_·5H_2_O, glucose, ethanol, agarose and NaOH were purchased from Sigma-Aldrich and used without further purification. Milli-Q water with a resistance of 18.2 MΩ was used for all experiments.

### Synthesis of Precursor Cu_2_O particles

Firstly, 1.25 g of CuSO_4_·5H_2_O was dissolved into 50 mL of H_2_O. The solution was continuously stirred at 55 °C for 2 min. Then 30 mL of NaOH solution (3 M) was rapidly poured into the CuSO_4_ solution, followed by quick heating to 70 °C. Blue Cu(OH)_2_ was immediately produced. After 5 min of stirring, 0.3 g of glucose was added into the suspension. The mixture solution was kept at 70 °C for 20 min. Red precipitate of Cu_2_O particles were formed. The particles were centrifuged and rinsed three times with water and ethanol.

### Synthesis of CuO hollow particles

0.08 g of the obtained Cu_2_O particles was dispersed into 3 mL of NaOH solution (0.1 M) via ultrasonication. The resulting suspension was continuously shaken for 24 h. The red Cu_2_O was gradually oxidized to black CuO. Then, the black CuO was collected and rinsed three times with water and ethanol.

### Preparation of photo-thermal coating

Firstly, 6 mg of CuO hollow particles and 10 mg of agarose were added into 1 mL of water, and was continuously stirred at 75 °C for 1 hour. Then, 30 μL of the mixture was drop-casted onto the exposed aluminium surface of the optical sensor head, and left to naturally dry.

## Additional Information

**How to cite this article**: Chen, G. Y. *et al*. Photodetector based on Vernier-Enhanced Fabry-Perot Interferometers with a Photo-Thermal Coating. *Sci. Rep.* **7**, 41895; doi: 10.1038/srep41895 (2017).

**Publisher's note:** Springer Nature remains neutral with regard to jurisdictional claims in published maps and institutional affiliations.

## Supplementary Material

Supplementary Information

## Figures and Tables

**Table 1:** Comparison of different photodetector technologies.

Material	Mechanism	Power sensitivity	Specific detectivity (D*)	Response time	Damage threshold	Detection area	Ref.
Polycrystalline silicon	Seebeck effect	202.8 V/W	2.85 × 10^8^ cm.√Hz/W	—	—	0.1 mm^2^	40
Bismuth/Antimony	Seebeck effect	290 V/W	8.8 × 10^8^ cm.√Hz/W	35 ms	—	0.20 mm^2^	41
B-doped poly Silicon-Gold	Seebeck effect	12.6 V/W	2.5 × 10^7^ cm.√Hz/W	5 ms	>5 W/cm^2^	0.28 mm^2^	42
AlGaAs/InGaAs	Seebeck effect	3000 V/W	1.4 × 10^8^ cm.√Hz/W	—	—	1.5 × 10^−3^ mm^2^	43
Copper oxide	Interferometry	0.76 nm/mW	2.2 × 10^5^ cm.√Hz/W	3 s	0.8 W/cm^2^	16 mm^2^	This work

## References

[b1] Zali R., Moravvej-Farshi M. K. & Abaeiani G. Internal photoemission-based photodetector on Si microring resonator. Opt. Lett. 37, 4925−4927 (2012). 23202092 10.1364/OL.37.004925

[b2] Patil V., Capone A., Strauf S. & Yang E. Improved photoresponse with enhanced photoelectric contribution in fully suspended graphene photodetectors. Sci. Rep. 3, 2791 (2013). 24071929 10.1038/srep02791 PMC3784941

[b3] Zhang G. . Highly efficient photovoltaic diode based on organic ultraviolet photodetector and the strong electroluminescence resulting from pure exciplex emission. Org. Electron. 10, 352−356 (2009).

[b4] Dennis P. J., Welch E. F., Alarie J. P., Ramsey J. M. & Jorgenson J. W. Development of a photothermal absorbance detector for use with microfluidic devices. Anal. Chem. 82, 4063−4071 (2010). 20411923 10.1021/ac902975r PMC2877626

[b5] Baylor D. A., Lamb T. D. & Yau K. W. Responses of retinal rods to single photons. J. Phsiol. 288, 613−634 (1979). PMC1281447 112243

[b6] Yuan H. . Polarization-sensitive broadband photodetector using a black phosphorus vertical p-n junction. Nat. Nanotechnol. 10, 707−713 (2015). 26030655 10.1038/nnano.2015.112

[b7] Dhanabalan S. C., Ponraj J. S., Zhang H. & Bao Q. Present perspectives of broadband photodetectors based on nanobelts, nanoribbons, nanosheets and the emerging 2D materials. Nanoscale. 8, 6410−6434 (2016). 26935809 10.1039/c5nr09111j

[b8] Huang Z. . Photoelectrochemical-type sunlight photodetector based on MoS_2_/graphene heterostructure. 2D Materials. 2, 035011 (2015).

[b9] Chen H., Liu H., Zhang Z., Hu K. & Fang X. Nanostructured photodetectors: from ultraviolet to terahertz. Adv. Mater. 28, 403−433 (2016). 26601617 10.1002/adma.201503534

[b10] Pelayo Garcia de Arquer F. & Konstantatos G. Large-area plasmonic-crystal-hot-electron-based photodetectors. ACS Photonics. 2, 950−957 (2015).

[b11] Rajan G. Introduction to optical fiber sensors in Optical fiber sensors: advanced techniques and applications 1−3 (CRC press, 2015).

[b12] Barnoski M. K. Introduction in Fundamentals of optical fiber communications 1−3 (Academic press, 1981).

[b13] High power thermal sensors. *Ophiropt datasheet*. http://www.ophiropt.com/laser-measurement/sites/default/files/10K-W-BB-45_30K-W-BB-74.pdf (2016).

[b14] Newport 919 series thermopile sensors. *Newport datasheet*. https://www.newport.com/medias/sys_master/images/images/ha6/h7a/8797036937246/919P-Thermopile-Detector-Datasheet.pdf (2016).

[b15] Wilson J. W., Stith J. J. & Stock L. V. A simple model of space radiation damage in GaAs solar cells. *NASA technical paper*. 2242c.1 (1983).

[b16] Chen G. Y., Zhang X., Brambilla G. & Newson T. P. Theoretical and experimental demonstrations of a microfiber-based flexural disc accelerometer. Opt. Lett. 36, 3669−3671 (2011). 21931427 10.1364/OL.36.003669

[b17] Chen G. Y. . Enhanced responsivity with skew mode excitation of transmission- and reflection-type refractometric sensors. Opt. Lett. 39, 3822−3825 (2014). 24978746 10.1364/OL.39.003822

[b18] Norland optical adhesive 61. *Norland datasheet*. https://www.norlandprod.com/adhesives/NOA%2061.html (2016).

[b19] Yoshino T., Kurosawa K., Itoh K. & Ose T. Fiber-optic Fabry-Perot interferometer and its sensor applications. IEEE T. Microw. Theory. 30, 1612−1621 (1982).

[b20] Lim D. . Enhanced photothermal effect of plasmonic nanoparticles coated with reduced graphene oxide. Nano Lett. 13, 4075−4079 (2013). 23899267 10.1021/nl4014315

[b21] Manikandan M., Hasan N. & Wu H. Platinum nanoparticles for the photothermal treatment of neuro 2A cancel cells. Biomaterials. 34, 5833−5842 (2013). 23642996 10.1016/j.biomaterials.2013.03.077

[b22] Au L. . A quantitative study on the photothermal effect of immune gold nanocages targeted to breast cancer cells. ACS Nano. 2, 1645−1652 (2008). 19206368 10.1021/nn800370j PMC2718847

[b23] Xu J. . Hierarchical CuO colloidosomes and their structure enhanced photothermal catalytic activity. J. Phys. Chem. C. 120, 12666−12672 (2016).

[b24] Wang L. . Optical absorption and photo-thermal conversion properties of CuO/H_2_O nanofluids. J. Nano. Sci. Nanotechno. 15, 3178−3181 (2015). 10.1166/jnn.2015.9657 26353558

[b25] Tian Q. . Hydrophilic flower-like CuS superstructures as an efficient 980 nm laser-driven photothermal agent for ablation of cancer cells. Advanced Materials. 23, 3542−3547 (2011). 21735487 10.1002/adma.201101295

[b26] Li M. . Inversed Vernier effect based single-mode laser emission in coupled microdisks. Sci. Rep. 5, 13682 (2015). 26330218 10.1038/srep13682 PMC4557034

[b27] Griffel G. Vernier effect in asymmetrical ring resonator arrays. IEEE. Photon. Technol. Lett. 12, 1642−1644 (2000).

[b28] Akcay C., Parrein P. & Rolland J. P. Estimation of longitudinal resolution in optical coherence imaging. Appl. Opt. 41, 5256−5262 (2002). 12211551 10.1364/ao.41.005256

[b29] Aketagawa M., Yashiki T., Kimura S. & Banh T. Q. Free spectral range measurement of Fabry Perot cavity using frequency modulation. Int. J. Precis. Eng. Man. 11, 851−856 (2010).

[b30] Bogaerts W. . Silicon microring resonators. Laser Photonics Rev. 6, 47−73 (2012).

[b31] Jewell J. M. Thermooptic coefficients of some standard reference material glasses. J. Am. Ceram. Soc. 74, 1689−1691 (2005).

[b32] Bouras N., Madjoubi M. A., Kolli M., Benterki S. & Hamidouche M. Thermal and mechanical characterization of borosilicate glass. Proc. JMSM. 2, 1135−1140 (2009).

[b33] Astheimer R. W. & Weiner S. Solid-backed evaporated thermopile radiation detectors. Appl. Opt. 3, 493−500 (1964).

[b34] Resbond high temperature ceramics. *isGroup datasheet*. http://www.isgroup-international.com/pdfs/Tapes_and_Adhesives_PDFs/tapes_and_adhesives_ceramic_adhesives.pdf (2016).

[b35] Holmes C., Gates J. C. & Smith P. R. Planarised optical fiber composite using flame hydrolysis deposition demonstrating an integrated FBG anemometer. Opt. Express. 22, 32150−32157 (2014). 25607179 10.1364/OE.22.032150

[b36] VIDRASA borosilicate physical and chemical properties. *VIDRASA datasheet*. http://www.vidrasa.com/eng/products/duran/duran_pf.html (2016).

[b37] Mattsson G. . Experimental evaluation of a thermopile detector with SU-8 membrane in a carbon dioxide meter setup. IEEE Sens. J. 9, 1633−1638 (2009).

[b38] Wolpert D. & Ampadu P. Temperature effects in semiconductors in Managing temperature effects in nanoscale adaptive systems 15–33 (Springer Science + Business Media, 2012).

[b39] Arbabzadah E. A., Shardlow P. C., Minassian A. & Damzen M. J. Pulse control in a Q-switched Nd:YVO_4_ bounce geometry laser using a secondary cavity. Opt. Lett. 39, 3437−3440 (2014). 24978505 10.1364/OL.39.003437

[b40] Zhou H., Kropelnicki P., Tsai J. M. & Lee C. Development of thermopile infrared sensor using stacked double polycrystalline silicon layers based on the CMOS process. J. Micromech. Microeng. 23, 065026 (2013).

[b41] Völklein F., Wiegand A. & Baier V. High-sensitivity radiation thermopiles made of Bi-Sb-Te films. Sensor. Actuat. A-Phys. 29, 87−91 (1991).

[b42] Choi H. & Wise K. D. A Silicon-thermopile-based infrared sensing array for use in automated manufacturing. IEEE T. Electron. Dev. 72−79 (1986).

[b43] Ang K. S. . AlGaAs/InGaAs thermopiles for infrared imaging using surface bulk micromachining technology. DTIP Barcelona 13672403 (2013).

