# Supplementary material for: Photodetector based on Vernier-Enhanced Fabry-Perot Interferometers with a Photo-Thermal Coating
Source: Sci Rep. 2017 Jan 31;7:41895. doi: 10.1038/srep41895 (PMC5282560; doi:10.1038/srep41895)
Supplement: Supplementary Information [file srep41895-s1.pdf]

Supplementary information

Photodetector Based on Vernier-Enhanced Fabry-Perot Interferometers with a Photo-Thermal Coating

George Y. Chen*,⊥,1, Xuan Wu⊥,2, Xiaokong Liu[2], David G. Lancester[1], Tanya M. Monro[1], and Haolan Xu*,2

[1] Laser Physics and Photonic Devices Laboratories, School of Engineering, University of South Australia, Mawson Lakes, South Australia 5095, Australia.

[2] Future Industries Institute, University of South Australia, Mawson Lakes, South Australia 5095, Australia.

*E-mail: george.chen@unisa.edu.au, haolan.xu@unisa.edu.au

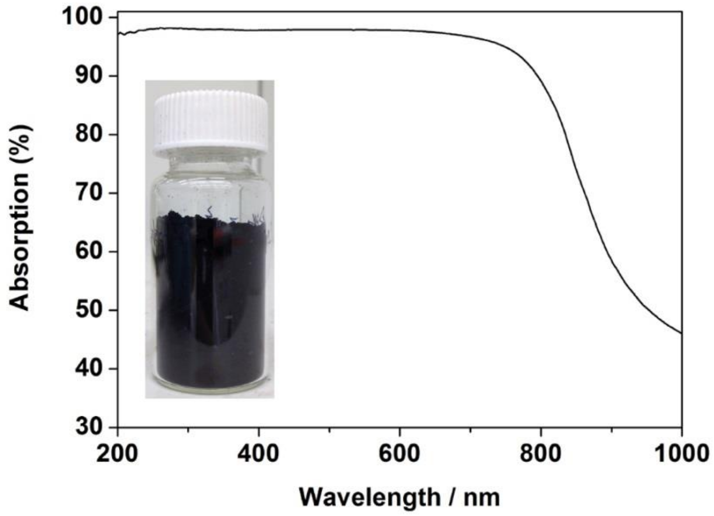

Figure S1. UV-Vis spectrum of flower-like CuO hollow micro-particles. Inset: the photograph of the synthesized particles which appears black in colour.

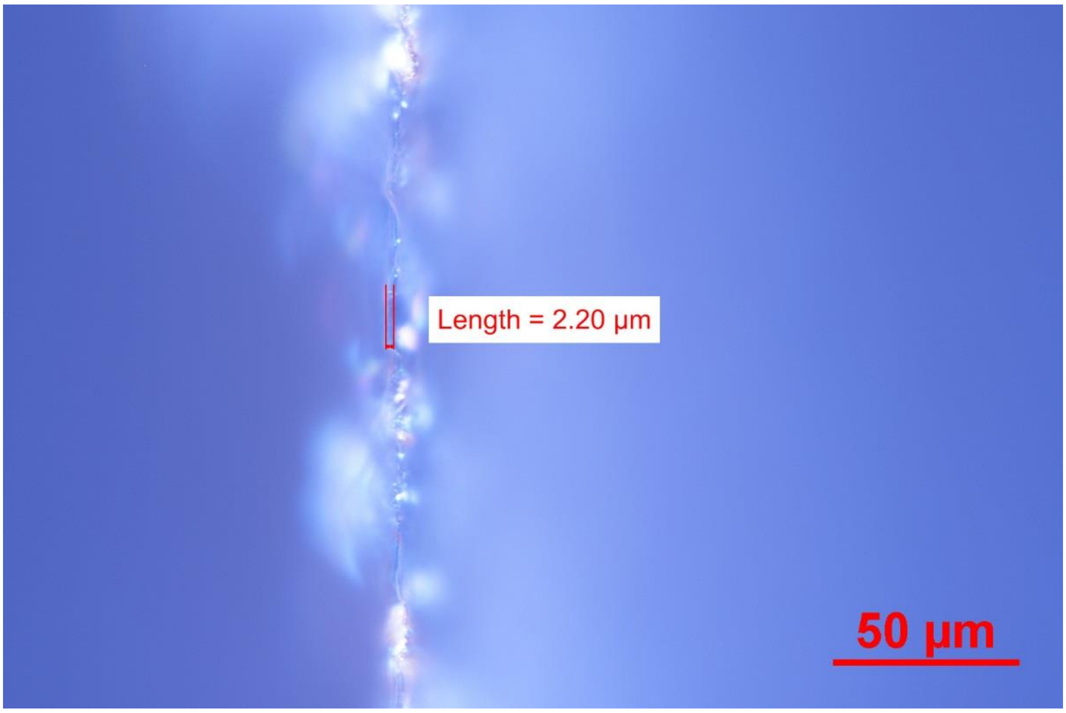

Figure S2. Microscope photograph showing the thickness of the photo-thermal coating consisting of CuO micro-particles embedded in hydrogel.

Table S1. Alternative photo-thermal materials.

Material	Photo-thermal performance	Cost
Gold nanoparticles [1]	High	$43.90 per g
Palladium nanoparticles [2]	High	$22.83 per g
Copper oxide (*this work*)	High	$0.0048 per g (copper)

Table S2. Alternative glass materials.

Material	Degradation temperature (˚C)	Transmission wavelengths (nm)
Fused silica [3]	1585	200-2600
Soda lime glass [4]	725	300-2600
Borosilicate [5] (*this work*)	815	330-2600

Table S3. Alternative epoxy materials.

Material	Degradation temperature (˚C)
Resbond ™ 931 [6]	2980
Duralco ™ 4703 [7]	343
NOA-61 [8] (*this work*)	260

References

1. Au, L. et al. A quantitative study on the photothermal effect of immune gold nanocages targeted to breast cancer cells. *ACS Nano.*, **2**, 1645-1652 (2008).
2. Manikandan, M.; Hasan, N.; and Wu, H. Platinum nanoparticles for the photothermal treatment of neuro 2A cancel cells. *Biomaterials.*, **34**, 5833-5842 (2013).
3. Corning 7980. *Corning datasheet*. http://www.sgpinc.com/corning.htm (2016).
4. Soda lime/AR/flint glass. *SGPINC datasheet*. http://www.sgpinc.com/sodalime.htm (2016).
5. Borofloat. *SGPINC datasheet*. http://www.sgpinc.com/borofloat.htm (2016).
6. Resbond high temperature ceramics. *isGroup datasheet*. http://www.isgroup-international.com/pdfs/Tapes_and_Adhesives_PDFs/tapes_and_adhesives_ceramic_adhesives.pdf (2016).
7. Ultra temperature epoxies. *Cotronics datasheet*. https://www.cotronics.com/vo/cotr/ea_ultratemp.htm (2016).
8. Norland optical adhesive 61. *Norland datasheet*. https://www.norlandprod.com/adhesives/NOA%2061.html (2016).
